# HIV-1 Vaccine Sequences Impact V1V2 Antibody Responses: A Comparison of Two Poxvirus Prime gp120 Boost Vaccine Regimens

**DOI:** 10.1038/s41598-020-57491-z

**Published:** 2020-02-07

**Authors:** Xiaoying Shen, Fatima Laher, Zoe Moodie, Arthur S. McMillan, Rachel L. Spreng, Peter B. Gilbert, Ying Huang, Nicole L. Yates, Nicole Grunenberg, M. Juliana McElrath, Mary Allen, Michael Pensiero, Vijay L. Mehra, Olivier Van Der Meeren, Susan W. Barnett, Sanjay Phogat, Glenda E. Gray, Linda-Gail Bekker, Lawrence Corey, Georgia D. Tomaras

**Affiliations:** 1 Duke Human Vaccine Institute, Duke University School of Medicine, Durham, North Carolina USA; 2 Perinatal HIV Research Unit, Faculty of Health Sciences, University of the Witwatersrand, Soweto, South Africa; 3 Vaccine and Infectious Disease Division, Fred Hutchinson Cancer Research Center, Seattle, Washington USA; 4 Division of AIDS, National Institute of Allergy and Infectious Diseases, National Institutes of Health, Bethesda, Maryland USA; 5 GSK Vaccines, Rixensart, Belgium; 6 GSK Vaccines (formerly Novartis Vaccines), Cambridge, Massachusetts USA; 7 Sanofi Pasteur, Swiftwater, Pennsylvania USA; 8 South African Medical Research Council, Cape Town, South Africa; 9 The Desmond Tutu HIV Centre, University of Cape Town, Cape Town, South Africa; 10 Department of Immunology, Duke University School of Medicine, Durham, North Carolina USA; 11 Department of Surgery, Duke University School of Medicine, Durham, North Carolina USA; 12 Department of Molecular Genetics and Microbiology, Duke University School of Medicine, Durham, North Carolina USA; 13 Present Address: Bill & Melinda Gates Foundation, Seattle, Washington USA

**Keywords:** HIV infections, Clinical trials, Translational research

## Abstract

In the RV144 trial, vaccine-induced V1V2 IgG correlated with decreased HIV-1 risk. We investigated circulating antibody specificities in two phase 1 poxvirus prime-protein boost clinical trials conducted in South Africa: HVTN 097 (subtype B/E) and HVTN 100 (subtype C). With cross-subtype peptide microarrays and multiplex binding assays, we probed the magnitude and breadth of circulating antibody responses to linear variable loop 2 (V2) and conformational V1V2 specificities. Antibodies targeting the linear V2 epitope, a correlate of decreased HIV-1 risk in RV144, were elicited up to 100% and 61% in HVTN 097 and HVTN 100, respectively. Despite higher magnitude of envelope-specific responses in HVTN 100 compared to HVTN 097 (p’s < 0.001), the magnitude and positivity for V2 linear epitope and V1V2 proteins were significantly lower in HVTN 100 compared to HVTN 097. Meanwhile, responses to other major linear epitopes including the variable 3 (V3) and constant 5 (C5) epitopes were higher in HVTN 100 compared to HVTN 097. Our data reveal substantial differences in the circulating antibody specificities induced by vaccination in these two canarypox prime-protein boost trials. Our findings suggest that the choice of viral sequences in prime-boost vaccine regimens, and potentially adjuvants and immunogen dose, influence the elicitation of V2-specific antibodies.

## Introduction

Most known correlates of protection for licensed vaccines involve antibody responses^1^. After the RV144 trial was identified as the first preventative HIV vaccine efficacy trial to report decreased HIV-1 acquisition^2^, advances were made in identifying correlates of risk. The heterologous prime-boost RV144 regimen, tested in Thailand, used a prime vaccine of a canarypox vector (ALVAC-HIV) with a subtype AE envelope (*env*) glycoprotein 120 (gp120) insert and a boost vaccine of subtype B/E bivalent gp120. The primary correlate of decreased HIV-1 risk was an IgG binding response to the variable loops 1 and 2 (V1V2) of the HIV envelope^3^. Secondary and exploratory correlates analyses revealed further evidence of V2-specific antibody immune correlates: linear V2 IgG^4^, V2 IgG breadth^5^, V1V2 IgG3^6^, and V1V2-specific complement binding antibodies^7^ correlated with decreased infection risk. A sieve analysis of breakthrough viral sequences from infected vaccine recipients and placebo recipients suggested vaccine-induced immune pressure at amino acid (aa) positions 169 and 181 in V2^8^.

Nonhuman primate studies conducted after RV144 supported the importance of non-neutralizing antibodies to V2 for protection from experimental challenge. Vaccine-elicited V2 IgG correlated with delayed SIV^9–13^ and SHIV acquisition^14^, and with viremia control after SIV infection^13^. Passive transfer of 830 A, a V2-specific monoclonal antibody (mAb), resulted in improved viremia control in nonhuman primates^15^.

Many studies have reported antiviral functions that include antibody Fc effector functions and direct neutralization mediated by antibodies that recognize V2^7,16–22^. V2-specific mAbs recognizing aa169, which were isolated from RV144 vaccine recipients, were shown to mediate tier 1 neutralization, bind to infectious virions^23^, and mediate killing of primary HIV-1 isolate infected cells^16^. Aa169 is located within the linear epitope sequence bound by RV144 vaccine-elicited antibodies that correlated with decreased HIV-1 risk^4^. The other known site of RV144-induced immune pressure in V2, aa181, is also part of the leucine-aspartic acid-isoleucine/valine (LDI/V) aa179–181 sequence motif reported to mediate HIV-1 envelope interaction with the gut mucosal homing integrin receptor α4β7 to facilitate cell-to-cell spread^19,20^. It has been postulated that V2-specific antibodies may block or interfere with the interaction between α4β7 and the HIV-1 envelope to prevent viral transmission^19,20^. In participants from the RV144 trial and the RV305 trial, in which delayed boosters were given to RV144 participants, a group of V2-specific mAbs were shown to be capable of blocking AE.92TH023 V2 peptide binding to α4β7^24^. The α4β7-blocking antibodies included both those targeting the linear V2 hotspot and those targeting the conformational epitopes^25^. V2-specific antibodies can mediate phagocytosis^18,26,27^ and can synergize with C1-C2 specific IgG for enhanced antibody-dependent cell-mediated cytotoxicity (ADCC)^28^. Furthermore, quaternary epitopes involving the V2 loop were identified as a target for broadly neutralizing antibodies^21,29^.

The Pox-Protein Public-Private Partnership (P5) program was established to develop a subtype C-directed vaccine based on the RV144 regimen^30^. As part of this program, two phase 1/2 trials were designed in South Africa: HIV Vaccine Trials Network (HVTN) 097^31^ (ClinicalTrials.gov NCT02109354) determined immune responses of an African population to the RV144 regimen, and HVTN 100^32^ (ClinicalTrials.gov NCT02404311) investigated the immunogenicity of the regimen adapted to subtype C. The selection criteria for the subtype C envelope immunogens included binding and affinity by V2-specific mAbs^33,34^. The HVTN 100 primary immunological data were evaluated against prespecified immunological criteria and guided the decision to proceed with the vaccine regimen into an efficacy trial, HVTN 702. Vaccine-elicited responses in HVTN 100 met all four prespecified go/no-go criteria for the continuation of the HVTN 702 trial, including the envelope-binding and V1V2-binding IgG response^32^.

Our study outlines the similarities and differences in the V2-directed responses elicited by the vaccines used in the HVTN 097 (also used in the RV144 trial) and HVTN 100 trials. We thereby demonstrate the importance of envelope sequence selection in optimizing epitope-specific antibody responses critical for vaccine protection.

## Results

### HVTN 097 and HVTN 100 vaccine trial designs

The HVTN 097 (phase 1)^31^ and HVTN 100 (phase 1–2)^32^ clinical trials both evaluated vaccine regimens consisting of a canary pox virus vector prime and a bivalent envelope gp120 protein boost in South Africa. Differences between the two regimens include: (1) the envelope vaccine strain was 92TH023 for the ALVAC prime and A244 and MN for the gp120 boost in HVTN 097, whereas HVTN 100 contained the ZM96 sequence for the ALVAC prime and 1086 and TV1 for the gp120 boost; (2) the adjuvant was aluminum hydroxide for HVTN 097 compared to MF59 for HVTN 100; (3) the protein dose in HVTN 097 was 3 times higher than that in HVTN 100; (4) the actual dose of ALVAC-HIV in HVTN 097 was 2.7 times more than that in HVTN 100 (ref. ^35^ and unpublished data); and (5) a 11-amino-acid (aa) sequence at the gp120 N terminus was removed and replaced with a herpes simplex virus (HSV) gD sequence for the gp120 proteins used as boosts in HVTN 097, whereas similar modifications were not made to the HVTN 100 gp120 constructs^33^.

For the comparison in our analysis, all immunological evaluations were performed on samples obtained 2 weeks after the second protein boost (6.5 months after the first ALVAC prime) in both trials.

### HVTN 100 elicited an overall higher magnitude of binding to gp120 antigens, but lower magnitude and breadth of binding to subtype C V1V2 antigens compared to HVTN 097

**Figure 1:**
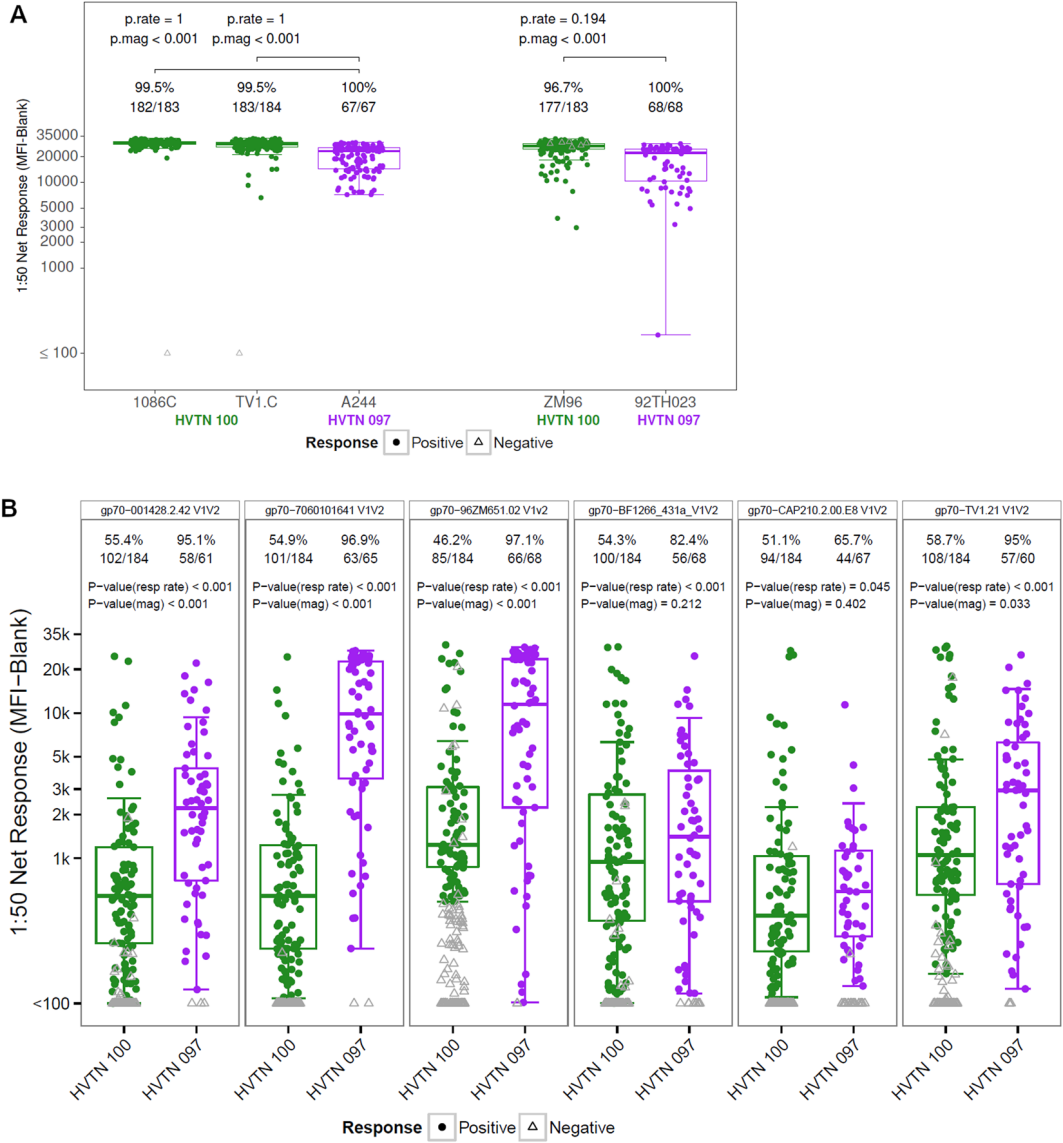
Higher gp120 and lower V1V2 responses in HVTN 100 compared to HVTN 097. Boxplots for magnitude of binding (based on positive responders only) to vaccine-matched gp120 antigens **(A)** and a panel of subtype C gp70 V1V2 antigens **(B**) in HVTN 100 (all available per-protocol participant, N = 185) and HVTN 097 (all available per-protocol participants, N = 73) measured in BAMA. Envelope antigens are indicated on top of each panel. Positive responders are shown as colored circles and negative responders as gray open triangles. Positivity rate and number of responders are listed on top of each box. Unadjusted p values for response rate (p.rate) and magnitude (p.mag) are listed on top of each panel. Data shown here are for a serum dilution of 1:50, which is lower than the 1:200 reported for HVTN 100 previously^32^ (thus higher antibody concentration) and the same as reported for HVTN 097 previously^35^.

We previously reported that HVTN 097 and HVTN 100 elicited a high magnitude of gp120 IgG responses^32,35^. Serum samples collected at 6.5 months (2 weeks after the second bivalent protein boost) were measured for binding response to gp120 antigens that match to the five subtype C and E HIV-1 envelope vaccine strains in HVTN 097 and HVTN 100 at the same 1:50 serum dilution (Fig. 1A). Response rates for gp120s were high (>95%) for both trials against the strain matched envelopes with no difference in response rates between the two trials. Of those HVTN 100 vaccinees with a positive antibody response, the magnitudes were significantly higher (p’s < 0.001, two-sided Wilcoxon rank-sum test) against vaccine-matched subtype C gp120 envelope proteins 1086C and TV1.C compared to HVTN 097 vaccinees with a positive response for the vaccine-matched subtype AE gp120 envelope protein A244.

We previously reported that subtype C V1V2 IgG responses were higher in magnitude and breadth in RV144 compared to HVTN 100^32^. Here we evaluate subtype C V1V2 IgG responses in HVTN 100 compared to HVTN 097. Sera from HVTN 100 and HVTN 097 vaccine recipients at 2 weeks after the second boost were evaluated for antibody binding to a panel of subtype C envelope V1V2 proteins (Fig. 1B). The V1V2 IgG response rate was higher in HVTN 097 compared to HVTN 100 with 5 out of the 6 V1V2 proteins reaching statistical significance (p < 0.001, two-sided Fisher’s exact test, Fig. 1B). The positive response rates for HVTN 097 ranged from 65.7% to 97.1% for the subtype C V1V2 proteins, in contrast to 46.2% to 58.7% for HVTN 100. Moreover, the magnitude of the V1V2 antibody response was higher in HVTN 097 compared to HVTN 100 with 3 of the 6 V1V2 proteins reaching statistical significance (p < 0.001, Fig. 1B, two-sided Wilcoxon rank-sum test).

### HVTN 097 elicited a higher magnitude and frequency of antibody responses to linear V2 epitope than HVTN 100

**Figure 2:**
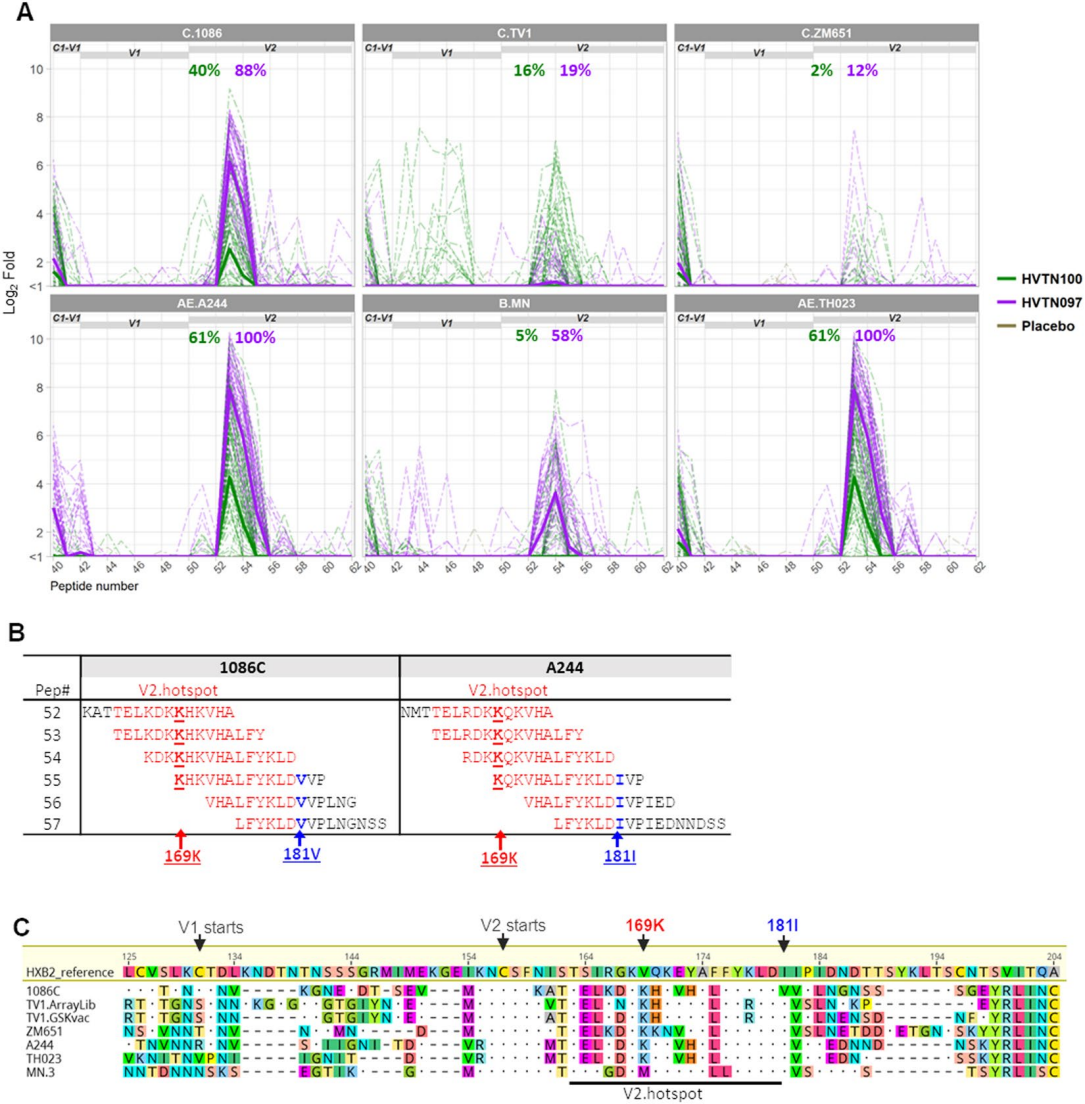
Higher V2 hotspot linear epitope binding in HVTN 097 compared to HVTN 097. Binding to linear V2 epitopes in HVTN 100 and HVTN 097 at 2 weeks post second protein boost measured by linear epitope mapping. (**A**) Magnitude of binding (log_2_ Fold post-/pre-immunization) to overlapping peptides in the V1V2 region of gp120. Thick solid lines represent weighted means (see Statistical Analysis) for all vaccine recipients in each study, and thin dashed lines represent individual vaccinee binding. Listed at the top of each plot are the positivity rates, color-coded for each group as indicated in plot legend, for binding to V2 hotspot. (**B**) Sequences for the overlapping peptides encompassing V2 hotspot, for C.1086 and AE.A244. (**C**) Alignment of V1V2 region of sequences for the 6 vaccine strains included in HVTN 100 and HVTN 097, with HXB2 sequence and numbering as reference. Sequences for TV1 included in array library (TV1.ArrayLib) and used as vaccine strain (TV1.GSKvac) are not identical and therefore both are included.

To elucidate the specificities and breadth of antibodies elicited in HVTN 097 and HVTN 100, we mapped the linear epitope binding specificities of serum samples from 2 weeks post second boost using a cross-subtype peptide library that included overlapping peptides for all 6 vaccine strains used in these two trials plus 7 consensus strains (evaluation of 2058 peptide sequences)^36^. A subset of 53 vaccine recipients and 5 placebo recipients in HVTN 100, and 45 vaccine recipients and 5 placebo recipients in HVTN 097 were selected for the analysis (see Methods for selection method and calculation of weighted means based on selection method). Binding to linear V2 peptides was observed in both studies, and was predominantly to subtype C 1086, subtype AE A244, and subtype AE 92TH023 peptides in both studies. Binding to the subtype B MN V2 peptides was also observed but only in HVTN 097 (Fig. 2A). The peak V2 region response spanned the peptide #53 sequence (for AE.A244, C.1086, and AE.92TH023) or the peptide #54 sequence (for B.MN) in the array library, corresponding to aa163-180 by the standard HXB2 numbering (Fig. 2A,B). This peptide region is of interest, since binding antibodies to this linear V2 hotspot correlated with decreased risk of HIV-1 infection in RV144^4^. Both the antibody magnitude and positivity rates to the V2 hotspot were higher in HVTN 097 compared to HVTN 100 (Fig. 2A, Table 1). The highest positivity rates were against the AE.A244 V2 hotspot peptides with 100% and 61% for HVTN 097 and HVTN 100, respectively. In contrast, positivity rates for C.1086 were lower in both studies compared to AE.A244, at 40% and 88%, respectively, for HVTN 100 and HVTN 097 (Fig. 2A). Differences in both magnitude and positivity rate of V2 hotspot binding between HVTN 100 and HVTN 097 were significant (p < 0.0001, HVTN 100 < HVTN 097, Wald test comparing mean magnitudes of all vaccine recipients or positive response rates via augmented inverse probability weighted estimating equations [see Statistical Analysis]) for AE.A244, AE.92TH023, B.MN and C.1086 (Table 1). Notably, AE.A244 and AE.92TH023 share identical amino acid sequence (Fig. 2C) for the V2 hotspot, and therefore the magnitude and positivity rates of the responses to V2 hotspot are identical for these two strains, as expected.

**Table 1:** Statistical summary and test result for linear epitope binding response comparison between HVTN 100 and HVTN 097.

Epitope	Strain	^a^Magnitude (Log_2_ Fold 6 mo/baseline)				^b^Positivity Rate			
		HVTN100	HVTN097	HVTN100-097	Raw.p	HVTN100	HVTN097	HVTN100-097	Raw.p
V2.hotspot	AE.A244; AE.TH023	4.28	7.93	−3.65	**<1.0E-16**	61%	100%	−39%	**<1.0E-16**
V2.hotspot	B.MN	0.58	3.68	−3.10	**<1.0E-16**	5%	58%	−53%	**6.88E-13**
V2.hotspot	C.1086	2.56	6.15	−3.60	**<1.0E-16**	40%	88%	−48%	**1.05E-10**
V2.hotspot	A.con	0.76	2.11	−1.36	**1.87E-05**	2%	25%	−23%	0.0005
V2.hotspot	AE.con	0.79	2.07	−1.28	**2.52E-05**	4%	28%	−24%	0.0003
V2.hotspot	AG.con	0.87	2.09	−1.22	**6.14E-05**	4%	23%	−19%	*0.0046*
C1.1	AE.A244	0.53	3.04	−2.51	**2.22E-16**	6%	52%	−46%	**1.45E-10**
C1.1	A.con; AE.con; AG.con	0.41	2.07	−1.65	**2.72E-08**	6%	43%	−37%	**3.09E-08**
C1.1	D.con	0.42	1.91	−1.49	**1.23E-07**	6%	40%	−34%	**1.78E-07**
C1.1	C.con; M.con	0.76	2.04	−1.28	**1.74E-07**	5%	29%	−25%	**1.15E-05**
C1.1	B.MN	0.50	2.09	−1.59	**4.25E-07**	6%	35%	−29%	**2.18E-06**
C1.1	AE.TH023	0.49	1.74	−1.25	**2.54E-06**	6%	34%	−28%	**3.74E-06**
C1.1	C.TV1; C.ZM651	0.69	1.97	−1.28	**1.26E-05**	11%	39%	−28%	**2.70E-05**
C1.1	B.con	0.53	1.65	−1.12	**3.68E-05**	6%	33%	−28%	**4.16E-06**
C1.2	A.con	1.50	3.25	−1.75	**6.66E-15**	21%	61%	−40%	**2.29E-08**
C1.2	C.TV1	3.83	4.31	−0.47	*0.0453*	56%	81%	−25%	**2.49E-05**
C1.2	AE.A244; AE.con; AE.TH023	4.17	4.53	−0.36	*0.1110*	67%	98%	−31%	**5.72E-13**
C1.2	AG.con; B.con; B.MN; D.con; M.con	4.00	4.13	−0.13	*0.5617*	63%	83%	−20%	0.0005
C1-V1	AE.A244	0.31	3.02	−2.71	**<1.0E-1**6	10%	53%	−43%	**5.68E-10**
C1-V1	C.TV1	0.79	1.00	−0.20	*0.3018*	11%	1%	10%	0.0006
C2.1	B.MN	0.20	0.87	−0.68	**8.09E-07**	1%	13%	−12%	0.0006
C2.1	C.con; C.TV1; C.ZM651	0.82	1.77	−0.95	**8.18E-06**	17%	39%	−22%	*0.0096*
C2.1	A.con; AG.con; B.con; M.con	0.28	0.91	−0.63	**1.61E-05**	9%	15%	−6%	*0.1715*
C2.1	AE.A244	1.25	2.24	−1.00	0.0003	16%	49%	−33%	**9.43E-06**
C2.1	AE.con; AE.TH023	1.25	2.24	−1.00	0.0003	25%	55%	−30%	**9.99E-05**
C2.1	C.1086	0.96	1.67	−0.71	0.0005	20%	22%	−2%	*0.7361*
C2.2	A.con	0.09	1.91	−1.82	**2.89E-15**	1%	23%	−23%	**1.92E-06**
C2.2	AE.A244; AE.con; AE.TH023; AG.con; C.1086; C.con; D.con; M.con	0.09	1.91	−1.82	**2.89E-15**	1%	42%	−41%	**2.18E-10**
C2.2	B.con; B.MN	0.09	1.91	−1.82	**2.89E-15**	1%	42%	−42%	**1.21E-10**
C2.2	C.TV1	0.09	1.91	−1.82	**2.89E-15**	1%	30%	−30%	**5.03E-08**
C2.2	C.ZM651	0.09	1.91	−1.82	**2.89E-15**	1%	21%	−21%	**5.86E-06**
C2.3	AG.con	6.61	2.65	3.95	**<1.0E-16**	87%	14%	73%	**<1.0E-16**
C2.3	B.con	2.25	0.63	1.62	**<1.0E-16**	15%	0%	15%	**1.89E-10**
C2.3	B.MN	6.46	3.83	2.63	**<1.0E-16**	94%	33%	61%	**<1.0E-16**
C2.3	C.TV1	7.45	4.16	3.29	**<1.0E-16**	94%	40%	54%	**4.66E-15**
C2.3	C.ZM651	6.31	4.24	2.08	**<1.0E-16**	90%	53%	37%	**1.22E-07**
C2.3	M.con	6.61	2.65	3.95	**<1.0E-16**	83%	16%	66%	**<1.0E-16**
C2.3	A.con	0.87	0.26	0.61	**1.42E-08**	4%	0%	4%	**8.19E-07**
C2.3	C.1086; C.con	1.01	1.42	−0.41	*0.0142*	29%	2%	27%	**1.63E-11**
C2.3	AE.A244; AE.con; AE.TH023	1.01	1.42	−0.41	*0.0142*	14%	0%	14%	**4.03E-09**
C2-V3	C.TV1	3.81	1.65	2.16	**3.13E-08**	37%	12%	25%	**4.32E-06**
C2-V3	C.con	0.20	0.08	0.12	0.0004	0%	0%	0%	NA
C2-V3	C.1086	0.11	0.01	0.10	*0.0031*	0%	0%	0%	NA
V3	A.con	8.15	4.74	3.41	**<1.0E-16**	100%	85%	15%	0.0001
V3	AG.con	8.86	6.39	2.47	**<1.0E-16**	100%	95%	5%	*0.0198*
V3	C.1086; C.con	9.19	6.27	2.92	**<1.0E-16**	100%	100%	0%	NA
V3	C.TV1	8.15	4.74	3.41	**<1.0E-16**	100%	96%	4%	*0.0296*
V3	M.con	7.36	5.41	1.96	**2.13E-14**	100%	84%	16%	*0.0154*
V3	B.con	6.29	4.85	1.44	**8.44E-14**	94%	94%	0%	*0.9769*
V3	B.MN	4.43	3.18	1.25	**5.43E-13**	94%	96%	−2%	*0.4080*
V3	C.ZM651	7.40	4.95	2.45	**6.87E-13**	100%	69%	31%	**6.84E-05**
V3	D.con	2.72	1.59	1.13	**2.45E-05**	47%	27%	20%	*0.0019*
V3	AE.TH023	6.83	5.60	1.24	**7.49E-05**	100%	84%	16%	*0.0129*
C4	A.con	3.08	1.08	2.00	**3.35E-14**	29%	5%	24%	**3.08E-09**
C4	B.con	1.96	0.86	1.10	**1.30E-05**	27%	8%	18%	**7.64E-05**
C4	B.MN	1.96	0.86	1.10	**1.30E-05**	16%	15%	1%	*0.8583*
C4	D.con	1.96	0.86	1.10	**1.30E-05**	27%	8%	19%	**6.98E-05**
C4	M.con	1.96	0.86	1.10	**1.30E-05**	29%	13%	16%	*0.0015*
C4	C.1086	0.33	0.10	0.23	**8.36E-05**	9%	5%	4%	*0.1916*
C4	C.TV1	0.23	0.07	0.16	**8.58E-05**	0%	4%	−4%	*0.0436*
C4	AE.con	0.44	0.15	0.30	0.0002	12%	0%	12%	**2.58E-05**
C4	AE.A244	0.44	0.15	0.30	0.0002	6%	13%	−7%	*0.0898*
C4	AE.TH023	0.44	0.15	0.30	0.0002	6%	9%	−3%	*0.4232*
C4	C.ZM651	0.83	0.68	0.14	*0.3342*	12%	2%	10%	**9.31E-05**
C4	AG.con	0.09	0.10	−0.02	*0.5222*	17%	0%	17%	**8.98E-09**
C5.1	AE.A244	4.74	3.03	1.71	**1.43E-08**	82%	57%	25%	0.0007
C5.1	AE.con; AE.TH023	5.34	4.04	1.30	**8.34E-06**	78%	76%	2%	*0.7705*
C5.1	C.con	4.96	3.85	1.11	0.0002	86%	72%	14%	*0.0474*
C5.1	A.con; AG.con; B.con; M.con	4.75	3.85	0.90	0.0003	78%	63%	15%	*0.0724*
C5.1	B.MN	4.75	3.85	0.90	0.0003	78%	57%	21%	*0.0078*
C5.1	C.1086; C.TV1	4.75	3.85	0.90	0.0003	89%	63%	26%	0.0009
C5.1	C.ZM651	4.75	3.85	0.90	0.0003	89%	76%	13%	*0.0622*
C5.1	D.con	4.75	3.85	0.90	0.0003	81%	61%	21%	*0.0098*
C5.2	C.con; C.TV1	8.06	3.04	5.02	**<1.0E-16**	100%	67%	33%	**5.08E-07**
C5.2	C.ZM651	4.95	2.08	2.87	**<1.0E-16**	87%	39%	48%	**6.37E-13**
C5.2	D.con	4.81	3.51	1.30	**1.27E-06**	99%	80%	19%	*0.0026*
C5.2	AE.A244	3.87	4.03	−0.15	*0.5731*	75%	94%	−18%	**3.00E-05**
C5.3	A.con	4.75	1.69	3.06	**<1.0E-16**	90%	33%	58%	**<1.0E-16**
C5.3	AE.A244	4.75	1.69	3.06	**<1.0E-16**	92%	43%	48%	**5.24E-13**
C5.3	AE.con; AE.TH023	4.84	1.61	3.23	**<1.0E-16**	93%	22%	71%	**<1.0E-16**
C5.3	AG.con	4.03	0.93	3.10	**<1.0E-16**	73%	15%	58%	**<1.0E-16**
C5.3	C.1086	4.39	0.71	3.68	**<1.0E-16**	59%	0%	59%	**<1.0E-16**
C5.3	C.ZM651	3.96	0.54	3.42	**<1.0E-16**	73%	8%	64%	**<1.0E-16**
C5.3	C.con	4.89	2.20	2.69	**2.00E-14**	98%	42%	57%	**<1.0E-16**
C5.3	C.TV1	4.89	2.20	2.69	**2.00E-14**	97%	44%	53%	**2.22E-16**
C5.3	M.con	4.35	2.21	2.14	**1.18E-11**	93%	44%	49%	**6.24E-14**
C5.3	D.con	4.41	2.12	2.29	**2.07E-11**	97%	46%	52%	**4.00E-15**
C5.3	B.MN	4.05	2.53	1.52	**4.04E-05**	90%	59%	32%	**3.79E-05**
C5.3	B.con	4.05	2.53	1.52	**4.04E-05**	80%	50%	31%	**5.56E-05**

Statistical significance considered at Raw p value < 0.001^a^.

^a^Only epitope-strain combinations with a p-value for either magnitude or for positivity < 0.001 are included in this table. A full table of the statistical testing results is available as Supplementary Table S1. Bold font: p < 0.0001; italic font: p > 0.001.

^b^Magnitudes and positivity rates are weighted means and adjusted positivity rates from augmented inverse probability weighting (See Statistical Analysis).

**Figure 3:**
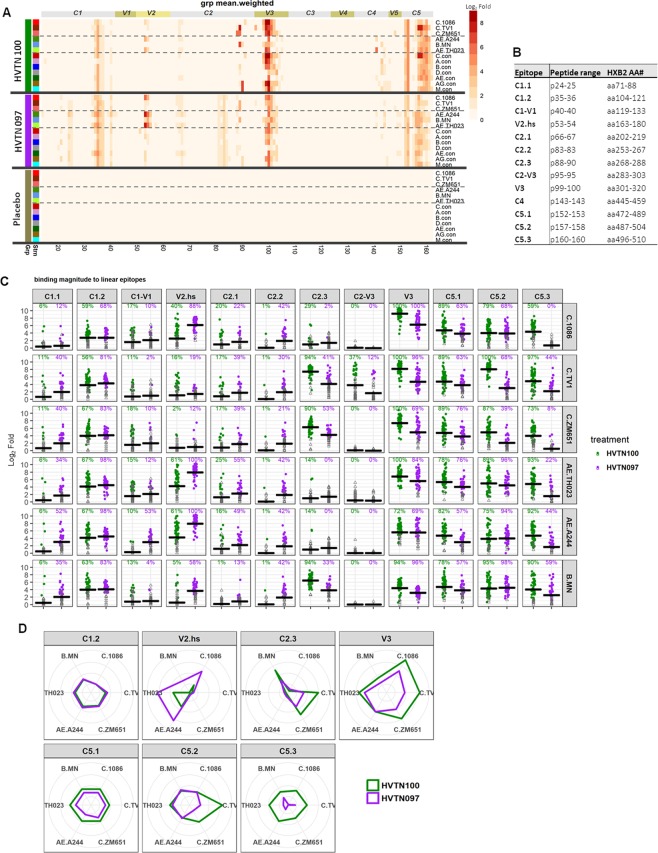
Different focuses of linear epitope specificity HVTN 100 versus HVTN 097. Linear specificities of antibodies elicited in HVTN 100 and HVTN 097 at 2 weeks post second protein boost characterized by linear epitope mapping. (**A**) Heatmap with weighted means (see Statistical Analysis) of binding magnitude (log_2_ fold post-/pre-immunization) comparing HVTN 100, HVTN 097, and placebo measured in linear epitope mapping. The peptide library used in the epitope mapping microarray contains overlapping peptides covering 13 consensus and viral envelope sequences as listed in the plot (also see Methods and Table S2). Thick black lines separate vaccine groups. Each row is for one strain, with dashed lines separating subtype C (top) and subtype B/E (middle) vaccine strains and consensus sequences (bottom). Constant and variable regions are labelled across the top, with the associated peptide number listed along the bottom. Minimum positivity cutoff is 1.58 (Log_2_ Fold) for individual samples. See Methods for positivity criteria. (**B**) Definition of linear epitopes identified. (**C**) Scatterplots for magnitude of binding to each epitope as indicated on top of each column. Magnitude of binding to each epitope is the highest binding magnitude to a single peptide within the epitope region as defined in 3B. Black cross bars represent weighted means (see Statistical Analysis) of all vaccine recipients. Closed circles indicate positive responses and open gray triangles indicate negative responses. Positivity rates (weight-adjusted, see Statistical Analysis) are indicated over the data points and color-coded accordingly. V2.hs- V2.hotspot. (**D**) Spider plot showing the breadth of binding (in coverage of the 6 vaccine strains) for each major epitope. Major epitope is defined as epitope with positivity rate >75% for any strain in either study. Labeled around each plot are the 6 vaccine strains, from the top clock-wise: C.1086, C.TV1, C.ZM651, AE.A244, AE.92TH023, and B.MN. Values plotted are weighted means (see Statistical Analysis) per study.

In addition to the antibody binding to the V2 hotspot against the vaccine strains C.1086, AE.A244, AE.92TH023, and B.MN, there was low magnitude V2 hotspot binding for several consensus strains (A.Con, AE.Con, AG.con) in HVTN 097, but this activity was absent for HVTN 100 (Table 1, Fig. 3A). These results indicate the presence of a broader V2 hotspot binding responses in HVTN 097 compared to HVTN 100.

### HVTN 100 elicited binding antibody responses to linear epitope specificities in gp120 that differed from HVTN 097

Linear gp120 epitope specificities of antibodies elicited in HVTN 100 and HVTN 097 were compared using data obtained from the peptide microarray described above, for serum samples at 2 weeks after the second protein boost. In both trials, antibody responses were directed to linear epitopes in C1, V2, C2, V3, and C5 regions across gp120 (Fig. 3A). While most of the linear epitopes recognized were similar in both studies, HVTN 097 alone targeted several epitopes in C1 and C2 though only at low magnitude. In HVTN 100, a strong V3 IgG response was dominant for all subtype C vaccine strains tested and for consensus sequences across subtypes A, B, C, AG and M (i.e., A.con, B.con, C.con, AG.con, and M.con). In addition, antibody responses to a C2 linear epitope were a dominant fraction of the responses against B.MN, and together with V3 were dominant for binding to C.ZM651, AG.con and M.con. C2, V3, and C5 specificities were dominant for binding to C.TV1, and C5 and V3 responses were co-dominant for binding to C.con. In HVTN 097, response against linear V2 dominated for AE.A244 and AE.TH23 and co-dominated with V3 response for C.1086, whereas for other strains, responses against linear epitopes in C1, V3, and C5 co-dominated (Fig. 3A).

We next compared the magnitude and positivity rates of antibody IgG binding to linear epitopes elicited in HVTN 100 and HVTN 097 (Fig. 3B,C). Significant differences were observed for multiple epitopes, with the most profound differences (p < 1 × 10^−16^ for either magnitude or positivity rate) seen for the V2 hotspot (multiple strains, HVTN 097 > HVTN 100), C1-V1 (AE.A244, HVTN 097 > HVTN 100), C2.3 (multiple strains, HVTN 097 > HVTN 100), V3 (multiple strains, HVTN 100 > HVTN 097), C5.2 (multiple subtype C strains, HVTN 100 > HVTN 097), and C5.3 (multiple strains, HVTN 100 > HVTN 097) (p < 0.001, Wald test comparing mean magnitudes of all vaccine recipients or positive response rates via augmented inverse probability weighted estimating equations [see Statistical Analysis]) (Table 1, Supplementary Table S1).

We further examined the relative magnitude and breadth of IgG binding to major epitopes. The major epitopes are defined as having a positivity rate >75% for any of the vaccine strains in either trial. As visualized by spider plots in Fig. 3D, magnitude and breadth of antibodies against V2 hotspot were relatively higher in HVTN 097 compared to HVTN 100. However, the magnitude and breadth for binding to C2.3 (C.TV1 and C.ZM651), V3 (especially for subtype C strains), C5.1, C5.2 (C.TV1 and C.ZM651), and C5.3 were relatively higher for HVTN 100 compared to HVTN 097. Overall, these data show substantial differences between these two poxvirus prime protein boost vaccine regimens with respect to the linear epitope specificities of the antibody responses elicited.

## Discussion

The promising results of the RV144 Thai vaccine efficacy trial prompted interest in optimizing the vaccine immunogen strategy to address the HIV epidemic in the most affected region of the world. Two major considerations guided further exploration of the poxvirus-protein prime-boost strategy in South Africa. First, it was not known whether the vaccine had to be modified to encode antigens from subtype C, the subtype of HIV most prevalent in sub-Saharan Africa where the epidemic is most marked. Second, it was not known whether the immune correlates identified in RV144 could be confirmed with another vaccine trial in a different population using a similar prime-boost regimen. Both conducted in South Africa, the HVTN 097 and HVTN 100 trials used the same strategy of a poxvirus vector-prime/protein-boost, but tested different HIV-1 envelope antigens expressed by the vector prime and administered as the gp120 protein boosts. In particular, the boosting antigens for HVTN 100 were derived from southern African subtype C HIV strains and selected based on their antigenicity, manufacturability, and preclinical immunogenicity with the goal to enhance immune coverage of genetically related strains in South Africa^33,34^. Immunogenicity evaluation two weeks after the 4th vaccination (second bivalent gp120 boost) timepoint in HVTN 100 revealed robust humoral responses by the subtype C ALVAC/bivalent gp120 vaccine^32^. HVTN 100 vaccine recipients developed IgG responses against gp120 antigens with sequences matched to the vaccine at a higher response rate and magnitude among positive responders compared to that in RV144, and a higher response rate for CD4+ T-cell response against the ALVAC insert strain compared to that in RV144. However, the response rate for the V1V2 region of 1086.C (one of the envelope strains in the protein boost of HVTN 100) was lower than that seen in RV144 against its vaccine sequence-matched AE.A244 V1V2. This raised questions as to whether the lower V1V2 responses observed in HVTN 100 compared to RV144 reflected a difference in participant populations and whether the different regimen subtype C ALVAC/gp120 immunogen in HVTN 100 elicited the same V2 specificities as did the subtype B/E regimen of RV144. In this analysis, we interrogated the specificity, magnitude, and breadth of HIV-1 envelope antibody responses in HVTN 097 and HVTN 100 to inform decisions on bridging of HIV vaccine candidates across subtypes with similar prime-boost strategies.

Consistent with previous findings of similar or stronger gp120-binding response in HVTN 100 compared to RV144^32^, we found significantly higher levels of gp120-binding responses to respective vaccine-matched antigens in HVTN 100 compared to HVTN 097. These data indicate that the overall immunogenicity of the subtype C HVTN 100 regimen was not inferior to that of the subtype B/E HVTN 097 regimen. However, binding to V1V2 scaffold protein antigens, including subtype C V1V2 antigens, were lower in HVTN 100 compared to HVTN 097. This is consistent with findings from previous comparisons of V1V2 responses between HVTN 100 versus HVTN 097 and RV144 where higher magnitude of binding to V1V2 proteins in HVTN 097 and RV144 compared to HVTN 100 was reported (^32^, Zhao, Fiore-Gartland, *et al.* PLoS One 2020, in press).

These results demonstrated that a V2-specific IgG response was elicited in both HVTN 100 and HVTN 097, and the same V2 linear epitope was targeted by both vaccines. However, both the magnitude and positivity of the linear V2 hotspot responses were lower in HVTN 100 compared to HVTN 097, even for subtype C strains. Positivity for linear V2 binding responses was highest for AE.A244 and AE.92TH023 in both studies, with positivity rates of 61% and 100% in HVTN 100 and HVTN 097 respectively. V2 hotspot antibodies elicited in HVTN 100 preferentially bound to AE.A244 V2 sequence over subtype C V2 linear sequences. Minimal binding responses were observed to the TV1 V2 linear epitope in HVTN 100 (16% positivity). The response rate to TV1 V1V2 gp70 scaffold was 58.5%. However, that included responses directed against V1, to which a relatively more frequent binding response was seen for TV1 strain compared to other strains in HVTN 100. Sequence alignment of the vaccine strains showed that C.1086 differs from AE.92TH023 by two amino acids in the V2 hotspot region and is closer to AE.A244 than any of the other strains tested in the array library for this epitope. This relatively high sequence similarity between the V2 hotspot sequences of C.1086 and AE.A244, the focusing of V2 hotspot binding response on these two strains in both trials, and the poor binding response to the C.TV1 V2 hotspot, suggest that of the three envelope immunogens in the HVTN 100 vaccine regimen, the C.1086 envelope contributed the most to the linear V2 responses. The higher magnitude of binding response to the AE.A244 V2 hotspot compared to the binding response to C.1086 V2 hotspot in HVTN 100 suggests that the AE.A244 V2 region is a better ligand for the V2 IgG elicited by either AE.A244 or C.1086 Env. Meanwhile, the higher binding responses to both the C.1086 and AE.A244 V2 hotspot in HVTN 097 compared to HVTN 100 indicate that AE.A244 Env is also a better immunogen than C.1086 Env for V2 specificity. The low antibody response to the C.TV1 V2 region in HVTN 100 suggests other subtype C vaccine candidates could improve upon the subtype C or cross-subtype V2 antibody responses observed in HVTN 100 by including antigens with more immunogenic V2 regions.

In addition to responses to the V2 hotspot, another potentially important anti-V2 response would be one that could interfere with α4β7 integrin binding. We hence looked for evidence of antibodies binding to peptides covering the LDI/V α4β7 binding site in V2. Peptide #57 in the microarray library is the peptide that centers around the LDI/V motif. Our data showed limited binding to this peptide in both HVTN 100 and HVTN 097 in only a very small subset of subjects, with positivity rates lower than 7.5% for any given strain by either trial. Therefore, antibodies directly targeting the linear LDI/V α4β7 binding site, as measured by the peptide microarray, are not likely to contribute significantly to potential protection mechanisms involving gp120-α4β7 interactions with these two vaccine regimens. Nevertheless, V2 hotspot binding mAbs isolated from RV144 and RV305 subjects were shown to be capable of blocking α4β7 binding^24,25^, and a more recent study^22^ showed that a group of V2 linear epitope binding antibodies (V2p)^26^ that can block α4β7 binding recognized helical V2 conformations involving a potential α4β7 binding secondary determinant at aa170-172^37^, which the V2 hotspot overlaps. Therefore, even though the binding response that directly targets the LDI/V motif was minimal in this study, other V2 antibodies including those targeting the linear V2 hotspot may have the potential to interfere with V2- α4β7 interactions.

Other than the difference in the sequences of the vaccine strains in the vector and protein immunogens for HVTN 100 and HVTN 097, the adjuvants and the protein doses differed as well: HVTN 097 used an inorganic adjuvant (aluminum hydroxide) while HVTN 100 used an organic adjuvant (MF59, an oil-in-water squalene-based emulsion with purported dose-sparing effect^38,39^); the protein doses in HVTN 100 were 1/3 that of the protein doses in HVTN 097; and, the ALVAC dose in HVTN 097 was 2.7 times the ALVAC dose in HVTN 100. However, the stronger or similar envelope gp120 responses in HVTN 100 compared to RV144 and HVTN 097 indicate that the lower V2 binding response in HVTN 100 is likely due to vaccine strain sequence differences rather than dose and adjuvant differences that tend to affect overall binding antibody responses. A previous study^40^ compared an ALVAC vCP1521 (AE.92TH023) prime, followed by different vaccine sequences as part of their bivalent B/E boosts. It included similar adjuvant and protein dose differences (alum vs. MF59, and 3-fold higher dose former vs. latter) as in our study. In contrast to our study, both Env gp120 and V1V2 IgG responses were higher in the alum adjuvanted study compared to the MF59 adjuvanted study. Therefore, the contrasting higher gp120 response and substantially lower V1V2 response reported in our study for HVTN 100 compared to HVTN 097 is likely due to virus sequence differences among the regimens instead of dose or adjuvant differences. Supporting this conclusion is that differences in antibody binding to other major HIV-1 envelope epitopes were in opposite directions from the V2 response (i.e., higher V3 and C5 binding in HVTN 100 compared to HVTN 097).

Further studies are underway to test additional subtype C strains that can complement the two sequences in the prime and boost, in an effort to improve the V2 response magnitude and coverage across subtype C strains (Korber, Haynes, personal communication). Furthermore, the stronger V2 response in HVTN 097 compared to HVTN 100, even against subtype C strains, suggests the potential of including subtype E antigens in vaccines for a subtype C epidemic for an enhanced V2 antibody response. Heterologous boosts incorporating a subtype E Env with demonstrated V2 immunogenicity (such as A244) and carefully selecting subtype C antigens for optimal V2 antibody responses may be worth further exploration for the subtype C epidemic in southern Africa. In addition, the V2 hotspot sequence is identical between the poxvirus prime strain AE.92TH023 and the protein boost strain AE.A244, making it a homologous boost for V2 in HVTN 097. In HVTN 100, such homology is absent between prime and boost strains, which could have contributed to the lower V2 response in HVTN 100. Interestingly, recent data examining the effect of delayed boosts of RV144 vaccine recipients in RV305 revealed preferential boosting of certain specificities including V2 by ALVAC 92TH023^41^. Therefore, similarity between prime and boost immunogens particularly for the V2 region should be considered when optimizing future vaccine regimens. Finally, HVTN 097 gp120 proteins included an 11-aa-deletion at the N-terminus of gp120, whereas HVTN 100 gp120 proteins do not have similar modifications. The N-terminal deletion in A244 has been shown to enhance the immunogenicity of the A244 gp120 in particular, including V1V2 IgG response^42^ while an 11-aa deletion at the N-terminus to other gp120 antigens, including C.1086, did not produce similar results. A hypothesis being investigated in the ongoing HVTN 702 efficacy trial is whether vaccine-elicited V1V2 IgG antibodies correlate with a decreased risk of HIV-1 infections in a subtype C region of the world. Ongoing phase 1 trials are testing different immunogen and adjuvant combinations toward the goal of identifying regimens that increase the breadth and/or magnitude of subtype C V2 region responses for consideration in future follow-up trials^30^.

In addition to the differences in elicitation of V2-specific antibodies, HVTN 100 and HVTN 097 also differ in the elicitation of antibodies to other linear epitope specificities. Even though the two vaccine trials targeted the same major regions of the HIV envelope (defined as epitopes with >75% positivity rate for any strain tested in either study), the magnitude and breadth of the antibody responses to specific sequences differed between trials. HVTN 097 elicited higher magnitude of C1.2 linear epitope binding and higher magnitude and breadth of binding to V2 hotspot whereas HVTN 100 elicited higher magnitude and breadth of binding to C5.1 and C5.3, and higher magnitude for subtype C strains for binding to C2.3 and C5.2. Overall, the highest mean magnitude (Log_2_ Fold) of IgG linear epitope responses in HVTN 100 was to V3 (9.2, for C.1086), C5.2 (8.1 for C.TV1), and C2.3 (8.8 for C.TV1). In contrast, for HVTN 097, the highest mean magnitude of IgG linear epitope responses was to V2 (7.9 for AE.A244) and V3 (6.4 for AG.con). Notably, this is consistent with our findings in nonhuman primate vaccine studies using HIV-1 envelope immunogens where V3 responses dominated in all studies tested except for one with AE.A244 gp120 protein as the immunogen boost^36^. Additionally, in both HVTN 100 and HVTN 097, antibodies targeting the linear V3 epitope showed cross-subtype breadth, with >65% positivity rate against all 6 vaccine strains in both studies, and >65% for the majority of the consensus strains. In contrast, antibodies targeting the linear V2 hotspot showed limited breadth, with positivity rates for AE.A244, AE.TH023, and C.1086 ranging from 40% to 61% for HVTN 100 and 88% to 100% for HVTN 097. Linear V2 response rates for the consensus sequences tested were all <10% for HVTN 100, and <30% for HVTN 097. The limited breadth of the V2 linear response highlights the challenge for vaccine coverage of diverse V2 sequences in regions with high HIV-1 envelope sequence diversity especially in the V2 region^43^.

One caveat of our analysis is that fine linear epitope mapping does not measure binding antibody responses against conformational or quaternary epitopes. Nevertheless, differences observed in linear specificity between HVTN 100 and HVTN 097 suggest different immunogenicity of the vaccine regimens. Further studies that examine differences in those non-linear epitopes should shed more light on the differences in immunogenicity of these subtype C and subtype B/E immunogens and vaccine regimens. Despite lower V2 response in HVTN 100 compared to HVTN 097, HVTN 100 elicited potent and cross-subtype binding antibody responses against HIV-1 envelope, with higher magnitude for multiple major epitopes compared to HVTN 097. Subsequent evaluation of the functions of the antibodies, including antibody Fc effector functions such as conformational C1-C2 antibody specificities that mediate ADCC^44^, elicited in these studies is needed to fully understand the differences in immunogenicity across these regimens. In addition, one of the objectives of testing RV144-like poxvirus-prime/protein-boost vaccines in South Africa is to see whether the correlates of decreased HIV-1 risk identified in RV144 can be repeated by subtype C vaccines. Our study found that the V3 response was higher for HVTN 100 compared to HVTN 097, indicating an opportunity to follow up on V3 IgG as a potential correlate of decreased HIV-1 acquisition risk^4,45^ in the HVTN 702 efficacy trial which uses the same vaccine regimen as HVTN 100^30^. However, the substantially lower V1V2 responses observed in HVTN 100 are a potential concern for the vaccine efficacy of the regimen. HVTN 702 will help address whether lower levels of the V1V2 correlate identified in RV144 translate to reduced vaccine efficacy.

In summary, our study revealed both quantitative and qualitative differences in the antibody responses elicited in HVTN 100 as compared to the HVTN 097 and RV144 clinical trials, particularly in the magnitude of the responses directed to a V2 hotspot epitope that correlated with a reduced risk of HIV-1 infection in R144 vaccine recipients^4^. Our data also demonstrated the substantial influence vaccine antigens can have on the elicited antibody specificities and underscores the need to select envelope sequences in the prime and boost immunogens targeted to the desired antibody specificities.

## Methods

### Ethics statement

#### HVTN 097 and HVTN 100 vaccine regimens

The HVTN 097 products and vaccine schedule were the same as that for RV144, and included ALVAC-HIV vCP1521 (expresses *env* gp120 of the subtype E strain 92TH023 linked with the gp41 transmembrane sequence of the subtype B LAI strain, as well as *gag* and *protease* from the subtype B LAI strain)(Sanofi Pasteur) at months 0 and 1, and ALVAC-HIV vCP1521 plus aluminum-hydroxide-adjuvanted bivalent B/E gp120 protein (A244 of the subtype E strain and MN from the subtype B strain) (Global Solutions for Infectious Diseases) at months 3 and 6^2^. Both MN and A244 gp120 constructs were made with an 11– amino acid deletion at the gp120 N-terminus and an HSV gD peptide tag inserted in place of the mutation^46^. The actual dose for ALVAC-HIV vCP1521 in HVTN 097 is 5.2 × 10^7^ CCID_50_^35^; and dose for gp120 proteins was 300 µg each protein, similar to that in RV144^2^.

The HVTN 100 regimen included ALVAC-HIV vCP2438 (expresses *env* gp120 of subtype C strain ZM96C linked with the gp41 transmembrane sequence of the subtype B LAI strain, as well as *gag* and *protease* from the subtype B LAI strain) (Sanofi Pasteur) at months 0 and 1, and ALVAC-HIV vCP2438 plus MF59-adjuvanted bivalent subtype C gp120 (of the C.1086 and C.TV1 subtype C strains)^33^ (GSK Vaccines) at months 3, 6, and 12^32^. The actual dose for ALVAC-HIV vCP2438 in HVTN 100 was 1.9 × 10^7^ CCID_50_ (unpublished data); and the dose for the gp120 proteins was 100 µg each protein.

The HVTN 097 trial was approved by the University of the Witwatersrand Human Research Ethics Committee for Klerksdorp and Soweto sites and by the University of Cape Town Ethics Committee for the Cape Town site^35^. The trial was registered with the United States National Institutes of Health Clinical Trials Registry (ClinicalTrials.gov NCT02109354) and the South African National Clinical Trials Registry (SANCTR number: DOH-27-0313-4201). The HVTN 100 trial was approved by the research ethics committees of the University of the Witwatersrand, the University of Cape Town, the University of KwaZulu-Natal, and the Medical Research Council^32^. Informed consent was obtained from all subjects in HVTN 097 and HVTN 100. All experimental methods were carried out in accordance with relevant guidelines and regulations.

#### Binding antibody multiplex assay (BAMA) against HIV-1 Env proteins and V1V2 scaffolds

Binding antibody multiplex assay was performed by a custom HIV BAMA as previously described^47^. Briefly, carboxylated fluorescent beads (Luminex Corp., Austin, TX) were covalently coupled to Env gp120 proteins and gp70-V1V2 scaffolds and were incubated with diluted serum samples. Antigen-specific IgG were detected with biotinylated goat anti-human IgG, followed by an incubation with streptavidin PE. Beads were then washed and acquired on a Bio-Plex instrument to measure florescence intensity. BAMA was run under GCLP compliant conditions, including tracking of positive controls by Levy-Jennings charts using 21CFR Part 11 compliant software. BAMA analysis for serum samples was performed on all HVTN 100 and HVTN 097 per-protocol (PP) vaccine recipients that have samples available for both 6.5 month and 13 month (N = 185 and 73 for HVTN 100 and HVTN 097, respectively). PP refers to receipt of the first four scheduled vaccinations and excludes those who had an HIV-1 positive test by month 6.5. HVTN 100 samples have been previously tested at a serum dilution of 1:200 and data were reported by Bekker *et al*.^32^. For a direct head-to-head comparison with HVTN 097, the HVTN 100 samples were tested again at 1:50 serum dilution. Differences among binding responses to antigens tested are consistent between the 1:50 and 1:200 dilution tests.

#### Linear epitope mapping for HIV Env

Linear epitope mapping of the purified IgG against HIV Env was performed as previously described with minor modifications^4,47^. Microarray slides were provided by JPT Peptide Technologies GmbH (Germany) by printing a library designed by Dr. B. Korber, Los Alamos National Laboratory, onto Epoxy glass slides (PolyAn GmbH, Germany). The library contains overlapping peptides (15-mers overlapping by 12) covering full length envelope sequences for seven consensus gp160 sequences and six gp120 virus strains. A full list of peptides in the microarray library is in Supplementary Table S2. Three identical subarrays, each containing the entire peptide library, were printed on every slide. All array slides were blocked and washed, incubated with 1:50 diluted plasma, followed by an incubation with AF647- conjugated goat anti-human IgG conjugated (Jackson ImmunoResearch, PA). Array slides were scanned at a wavelength of 635 nm with an InnoScan 710 AL scanner (Innopsys, Carbonne, FRANCE) using XDR mode. Images were analyzed using Mapix 8.0 software to obtain fluorescence intensity values for all peptides. Array data were then processed using R package pepStat. Magnitude of binding is defined as: Signal = log2-transformed fold difference in intensity values between each post-immunization sample and its baseline (matched baseline sample or median of all baseline samples, whichever is higher). Positivity call is made for every post-immunization sample against every peptide with the following positivity criteria: (1) fluorescence intensity of the post-immunization sample against the peptide >95^th^ percentile intensity of all pre-immunization samples for the peptide; and (2) signal for the peptide >1.58, which represents a 3-fold difference in fluorescence intensity between sample and its baseline. For positivity determination, pepStat process included the “smooth” function to reduce false positivity. Since linear epitope mapping for RV144 was performed over 10 years ago and the assay and analytical methods have been updated, the positivity rates cannot be directly compared to RV144. Tests for HVTN 100 and HVTN 097 were performed contemporaneously for the immunogenicity comparison in order to ensure the robustness of the comparison between the two trials.

#### Selection of subjects for linear epitope mapping

For the detailed epitope mapping by peptide microarray, we focused on vaccine recipients with detectable antibody responses to the V1V2 region. Plasma samples were down-selected based on having sufficient levels of antibody binding while maintaining gender balance. The details for each protocol based on pre-specified binding criteria are described here. For HVTN 100, a subset of 53 PP vaccinees and 5 placebos were selected for linear epitope mapping based on binding plasma IgG against V1V2 antigens as measured in BAMA as follows: 1) Select the n = 32 PP vaccinees with the highest IgG binding antibody titers (log10 net MFI > 2.75) to three V1V2 antigens (subtype B gp70_B.CaseA_V1_V2, subtype C C.1086C_V1_V2_Tags, and subtype C gp70-TV1.21 V1V2). 2) Select an additional n = 6 of the highest PP vaccinee responders to C.1086C_V1_V2_Tags whowere not high responders to gp70_B.CaseA_V1_V2 and/or subtype C gp70-TV1.21 V1V2 (i.e., log10 net MFI < =2.75). 3) Select a random sample of n = 15 from the remaining PP vaccinees, stratified by gender to balance the gender ratio observed in the n = 38 high responders (39% female:61% male). 4) Randomly select n = 5 PP placebos, stratified by gender.

For HVTN 097, a subset of 45 PP vaccinees and 5 placebos were selected for linear epitope mapping based on binding plasma IgG against V1V2 antigens as measured in BAMA as follows: 1) Randomly select n = 30 PP vaccinees with the highest visit 14 IgG binding antibody titers (log10 net MFI > 2.75) to all three primary antigens (subtype B gp70_B.CaseA_V1_V2, subtype C gp70-TV1.21 V1V2, and subtype AE AE.A244 V1V2 Tags) based on the two (of three) available antigens used in the HVTN 100 selection and the V1V2 antigen with the strongest response, while maintaining gender balance across the entire selection and treatment group (T1/T2) balance between step 1 and step 2. 2) Select a random sample of n = 15 from the remaining PP vaccinees not among the high responders as defined in step 1, stratified by gender to balance the gender across the entire selection and treatment group (T1/T2) balance between step 1 and step 2. 3) Randomly select n = 5 PP placebos, stratified by gender. These pre-specified selection criteria were met before initiation of the epitope mapping by peptide microarray.

### Statistical analysis

Comparisons of magnitude and positivity of antibody responses for V1V2 protein binding in BAMA were performed using a two-sided Wilcoxon rank-sum test (for magnitude of positive responders) and a two-sided Fisher’s exact test (for positive response rates) respectively, using SAS (version 9.4; SAS Institute, Cary, NC, USA) and R statistical software (version 3.3.2; R Foundation for Statistical Computing, Vienna, Austria). P-values less than 0.001 were considered significant; no adjustment was made for multiple testing.

For linear epitope mapping data, the subset of subjects included in the analysis were randomly selected with sampling probabilities depending on participant variables (see “Selection of subjects for linear epitope mapping” under Methods). To account for this subset analysis, augmented inverse probability weighting^48^ based on the corresponding selection criteria was performed for estimation and hypothesis testing about response rates, mean magnitudes, and any other population-level parameters that were estimated for data visualization, using R statistical software (version 3.5.1; R Foundation for Statistical Computing, Vienna, Austria). This method yields estimates of means, mean differences, 95% confidence intervals for mean differences, and 2-sided Wald-based p-values for whether a mean difference departs from zero. Analysis was adjusted for potential confounding by gender.

## Supplementary information

Supplementary Table 1.

Supplementary Table 2.

## Data Availability

All data generated or analyzed during this study are included in this published article (and its Supplementary Information files) or are available at the following link https://atlas.scharp.org/cpas/project/HVTN%20Public%20Data/Cross-Protocol%20HVTN%20Manuscripts/begin.view?.
